# Olympic distance duathlon and cardiac performance in highly‐trained triathletes

**DOI:** 10.14814/phy2.70154

**Published:** 2024-12-26

**Authors:** J. A. Donaldson, J. D. Wiles, M. Papadakis, S. Sharma, R. Sharma, J. M. O'Driscoll

**Affiliations:** ^1^ Department of Cardiovascular Sciences, College of Life Sciences, Cardiovascular Research Science Glenfield Hospital Leicester UK; ^2^ School of Psychology and Life Sciences Canterbury Christ Church University Kent UK; ^3^ Cardiology Clinical Academic Group, St George's University of London London UK; ^4^ Department of Cardiology St George's University Hospitals NHS Foundation Trust London UK; ^5^ Diabetes Research Centre, College of Life Sciences University of Leicester Leicester UK

**Keywords:** cardiac autonomics, duathlon, myocardial performance, troponin

## Abstract

The effects of triathlon exercise on cardiac function are well documented. While Olympic triathlon (swim‐bike‐run) remains the standard format, increasing concerns about water quality in natural waterways present ongoing challenges for open‐water swimming events, highlighting the potential need to consider alternative formats such as duathlon (run‐bike‐run) in some circumstances. An additional run may increase the overall metabolic and cardiovascular demand compared with the swim in triathlon, leading to reduced future performance. Conversely, the majority of EICF research reports reversal of post‐exercise perturbations within 24–7 days of recovery but duathlon has not yet been studied in this context. Therefore, this study aimed to investigate the cardiac, autonomic, haemodynamic and biomarker responses during and following two Olympic distance (OD) duathlon separated by 7 days of recovery. Highly‐trained (*V* O_2max_ >60 mL·kg^−1^·min^−1^) male participants (*n* = 10) completed two lab‐based OD duathlons, either continuous (BD) or with functional measurements after each leg (UD), separated by 7 days of rest. Conventional echocardiography recorded standard and tissue Doppler measures of left ventricular (LV) structure and function. Speckle tracking echocardiography was used to measure global longitudinal strain (GLS). Time and frequency domain analysis of HRV, as well as plasma high sensitivity cardiac troponin T (hs‐cTnT) were measured pre and post exercise. In the broken duathlon trial (BD) cardiac measurements and blood samples were also taken between each leg. In the unbroken duathlon (UD) participants performed each leg sequentially. Duathlon exercise resulted in similar cardiac functional and biomarker alterations as previously reported in triathlon and standalone running and cycling exercise. Cardiac troponins were still elevated following 24 h^−1^ of recovery. However, functional changes were resolved within 24 h^−1^ of passive recovery and did not impair subsequent duathlon performance, or pre‐exercise measurements 7 days after the first trial. Whether or not elite or recreational athletes experience the same magnitude and reversibility of these changes remains to be elucidated further.

## 1 BACKGROUND

Transient perturbations in cardiac function measured by echocardiography, autonomic modulation, and cardiac biomarker release following strenuous exercise have been observed in athletic and non‐athletic populations (Lord et al., 2018; Stewart et al., 2016). The immediate exercise induced cardiac functional changes (EICF) may resemble those observed in patients with heart disease (La Gerche et al., 2012), and there is evidence that a lifetime of endurance athleticism causes long term structural changes to the heart and increases the life‐time risk of cardiomyopathy (Franklin et al., 2020) or atrial fibrillation (Newman et al., 2021). Alternatively, the physiological and mechanical stress caused by prolonged strenuous exercise (PSE) may provide stimulus for cardiomyocyte adaptation, similar to the changes seen in skeletal muscle following exercise training (Clarkson et al., 1992; Shave et al., 2004). The cardiovascular response to PSE is multifaceted and there is huge inter‐individual variation among the cardiac parameters studied (Donaldson et al., 2019). Individual fitness and training experience likely plays a significant role in the magnitude of EICF with a previous meta‐analysis demonstrating that an individual's years of training history as a significant predictor of cardiac damage biomarker release (Shave, Baggish, et al., 2010). Further, the majority of EICF research that reports post‐exercise cardiac ‘fatigue’ and performed follow‐up measures within 1–7 days after the onset of exercise typically report a return to pre‐exercise conditions (Oxborough et al., 2010).

There has been substantial investigation into the impact of the duration and the intensity of exercise that provokes transient reductions in cardiac function, autonomic modulation and the release of biomarkers, such as cardiac troponin (cTn) (Beaumont et al., 2017; Coates et al., 2023; Donaldson et al., 2019; Kleinnibbelink, Hulshof, et al., 2021). Research has primarily focused on long‐duration (>3 h) endurance exercise. However, more recent works have demonstrated progressive reduction of both left ventricular (LV) and right ventricular (RV) mechanics, and elevations in cTn following bouts of shorter duration (30–120 min^−1^), high intensity (mean HR >170 b∙min^−1^) exercise (Kleinnibbelink, van Dijk, et al., 2021; Shave, Ross, et al., 2010; Stewart et al., 2015), which may have implications for recreational and elite athletes competing in events of this duration range, and for athletes training and competing multiple times within a weekly period.

Duathlon (run, cycle, run) is a popular off‐season alternative to triathlon competition (Nikolaidis et al., 2021), with the Olympic distance consisting of a 10‐kilometer (km) run, 40 km cycle and 5 km run, and is currently under‐studied within the field of cardiac fatigue. Duathlon may incur greater levels of skeletal muscle and cardiac fatigue due to the substitution of the non‐weight bearing swim for an additional run leg (Millet et al., 2009). The repeated eccentric muscle contractions during running lead to increased mechanical stress and microtrauma in the lower limbs, triggering inflammatory responses and elevated systemic stress markers. This mechanical loading, combined with prolonged cardiovascular strain, can amplify both peripheral and central fatigue mechanisms. The thermoregulatory (Sparks et al., 2005), metabolic (Berry et al., 2016) and endocrine (Alvero‐Cruz et al., 2011) responses to duathlon performance have been previously investigated which demonstrate the impact on these systems is similar to triathlon performance. Duathlons are increasingly utilized as an alternative to triathlon when environmental conditions affect water safety or quality. Rising global temperatures and changing weather patterns can impact water conditions through increased algal blooms, storm water runoff, and altered seasonal precipitation patterns (Moore et al., 2008) potentially leading to more frequent requirements for duathlon alternatives. This adaptation in race format represents a significant shift in physiological demands for athletes, as replacing the non‐weight‐bearing swim segment with running fundamentally alters the biomechanical and cardiovascular stress of the event. The growing frequency of such format changes due to environmental challenges highlights the importance of understanding the specific physiological impacts of duathlon performance, particularly given that athletes must often adapt their race strategy with limited notice. Despite this increasing relevance, there has been limited research examining the acute physiological responses to duathlon competition, particularly regarding cardiovascular function. Accordingly, we aimed to investigate the effects of duathlon performance on cardiac function, biomarker release, and autonomic modulation. As an individual's fitness and training history likely play a role in the development of EICF, and to best understand any effects on these variables in a population closely resembling an elite cohort, we recruited participants who's *V* O_2max_ exceeded 60 mL kg min^−1^ and who had been competing and racing in triathlon for a period >3 years. The participants performed two OD duathlons, separated by 7 days of recovery to test whether participation in strenuous duathlon racing resulted in decrements to cardiac function as well as overall exercise performance as a result of lingering cardiac functional perturbations.

## 2 METHODS

### 2.1 Study protocol and ethics

All procedures for this investigation conformed to the Declaration of Helsinki principles and Canterbury Christ Church Universities Faculty of Social and Applied Sciences Research Ethics Committee approved the study. Signed, informed written consent was obtained from all participants. Participants were recruited through social media and word of mouth at triathlon, running, swimming, and cycling clubs local to the Canterbury (UK) area. Requisites for recruitment to the study were at least 3 years of multisport (triathlon, duathlon) training and racing experience, male, to be under 40 years of age, and to achieve a *V* ^⋅^O_2max_ (relative) of at least 60 mL∙kg^−1^∙min^−1^ during either a run or cycle cardiopulmonary exercise test (CPET). Participants reported no medication use and were actively training for and competing in triathlon and duathlon competitions at the time of participation in the study. In addition, pre‐participation health screening according to ACSM guidelines ensured that participants were apparently healthy, non‐smokers and had no contraindications to exercise (Thompson et al., 2013). Participant demographic and exercise characteristics are displayed in Table 1.

**TABLE 1:** Participant characteristics.

Measure	Unit	Mean	SD
Stature	cm	179.1	4.5
Mass	Kg	73.4	6.9
Age	Years	32.6	5.9
History_TX_	Years	10.6	6.0
Bike			
*V* ^⋅^O_2max_ (Relative)	mL∙kg^−1^∙min^−1^	60.3	3.70
*V* ^⋅^O_2max_ (Absolute)	L∙min^−1^	4.42	0.35
*V* ^⋅^E_MAX_	L∙min^−1^	160.7	17.4
P*V* ^⋅^O_2max_	W	368.5	28.8
HR_MAX_	b∙min^−1^	177.9	7.61
Run			
*V* ^⋅^O_2max_ (Relative)	mL∙kg^−1^∙min^−1^	61.2	5.89
*V* ^⋅^O_2max_ (Absolute)	L∙min^−1^	4.54	0.38
*V* ^⋅^E_MAX_	L∙min^−1^	173.8	15.5
v*V* ^⋅^O_2max_	km∙h^−1^	18.8	0.79
_tLim_v*V* ^⋅^O_2max_	s^−1^	49.3	10.2
HR_MAX_	b∙min^−1^	183.8	10.8

*Note*: HistoryTX = number of years training for endurance sports, VEMAX = maximum minute ventilation (*V* ^⋅^E), P*V* ^⋅^O_2max_ = power output (cycling) at *V* ^⋅^O_2max_, v*V* ^⋅^O_2max_ = running velocity at *V* ^⋅^O_2max_, tLimv*V* ^⋅^O_2max_ = time spent at *V* ^⋅^O_2max_ at the end of the CPET test.

### 2.2 Study design

All participants reported to the laboratory at Canterbury Christ Church University (Section of Sport and Exercise Sciences in Canterbury, Kent, UK) on 6 separate occasions, and abstained from caffeine before each visit, and exercise for a minimum of 48 h^−1^ before each experimental trial. During the first two visits, participants performed pre‐participation health screening and exercise testing, which involved a run and bike CPET, the order of which was randomized. The CPET trial consisted of baseline measurements for HRV, transthoracic echocardiogram (TTE), anthropometric and demographic characteristics (see Table 1) followed by an incremental exercise test. The bike CPET was performed on an SRM cycling ergometer (SRM, Germany) with increments of 25 W from a 100 W starting point. The run CPET was performed on a motorized treadmill (Woodway ELG, Woodway, Wisconsin, USA) with increments of 1.0 km∙h^−1^ from a 10 km∙h^−1^ initial stage. Oxygen uptake (*V* ^⋅^O_2_), carbon dioxide output (*V* ^⋅^CO_2_) and minute ventilation (*V* ^⋅^E) were measured breath‐by‐breath using an automated metabolic measurement system (Jaeger Oxycon Pro, Carefusion, Pennsylvania, USA), and were subsequently averaged over 30‐s intervals. Peak exercise values were calculated as the average of the highest 30‐s value attained during the CPET. Following the CPET, TTE, HRV and body mass measurements were immediately recorded.

**FIGURE 1:**
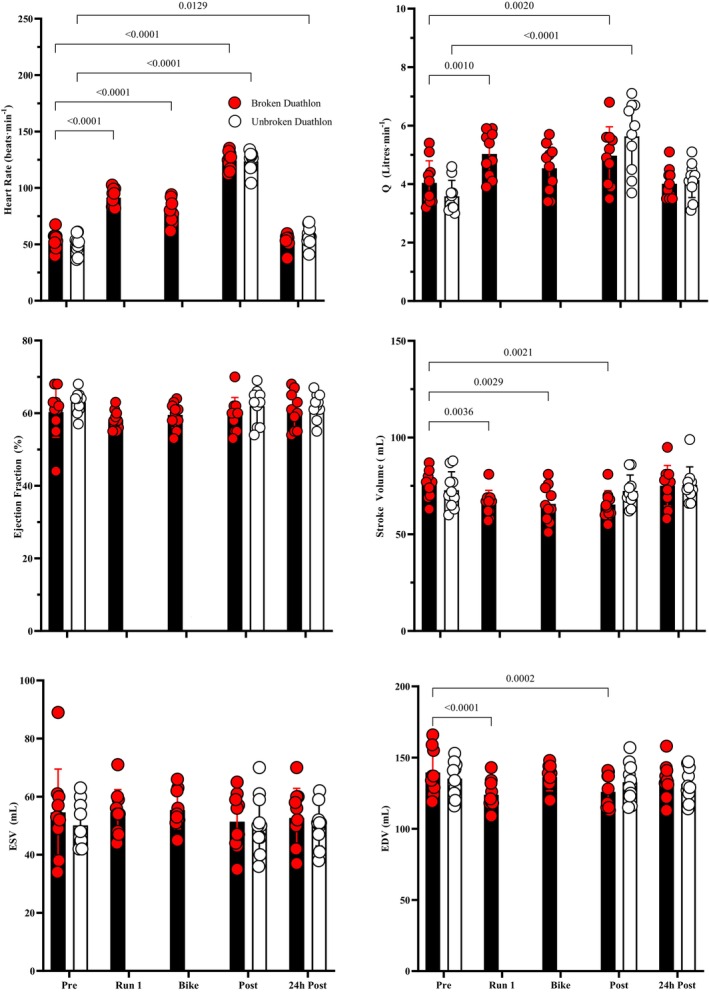
Plot of left ventricular systolic functional parameters following each stage of the BD (closed bars) and UD (open bars) trials. *n* = 10 participants. Results of the paired‐samples *t*‐tests results are presented on the figure where significant (*p* = 0.05) differences from the pre‐exercise values within each trial occurred. Individual data points are represented by red circles for the BD trials and open circles for the UD trials.

During the subsequent experimental trials, participants reported to the laboratory for baseline measurements, and venous blood samples were also taken. Participants then performed either a continuous unbroken duathlon (UD) trial or discontinuous broken duathlon (BD) trial, the order of which was randomized between participants using an online random number generator (Haahr, 2021). Each duathlon trial involved a laboratory‐based 10 k run, 40 k cycle, and 5 k run using the same equipment as the CPET. In the UD trial, participants were given a controlled 5 min^−1^ ‘transition’ time between legs to change clothing and footwear as needed. Between legs in the BD trial, HRV, TTE, blood sampling, and body mass measurements were performed (Figure 1). The average time for the intra‐leg measurements was 35 ± 10 min^−1^ between each leg and at the end of the trial following the second run. Participants were encouraged to replenish lost bodily fluids at an assumed sweat‐loss rate of 1 L∙h^−1^ to ensure euhydration throughout the trials (González‐Alonso et al., 2008). Following 24 h of passive recovery, participants returned to the laboratory for subsequent baseline measurements and blood sampling. Participants returned to the laboratory and repeated the remaining trial 7 days after the completion of the first duathlon.

### 2.3 Echocardiography

Resting TTE was performed in the left lateral decubitus position by one highly experienced sonographer using a VividQ ultrasound machine (GE Medical, Horton, Norway), equipped with a 1.5–4.5 MHz transducer. Images were stored in raw digital imaging and communication in medicine (DICOM) 158 format and were exported to an offline workstation (EchoPac, version 113 GE Medical, Horton, Norway). Data‐analysis, from three stored cycles, was performed by a single observer with experience in echocardiography using commercially available software (EchoPac, version 113, GE Medical, Horton, Norway). The observer was blinded for the timing (pre vs. post‐exercise) and condition (broken vs. unbroken duathlon) under which echocardiography was performed.

#### 2.3.1 Conventional measurements

Cardiac structural and functional measurements were made according to the current guidelines for cardiac chamber quantification from the ASE and EACI (Lang et al., 2015). The following LV functional measures were determined: end diastolic volume (EDV), end‐systolic volume (ESV), modified Simpson's biplane left ventricular ejection fraction (EF), from which stroke volume (SV), and cardiac output (Q̇) were calculated, tissue Doppler imaging (TDI) of the mitral annulus (LV S′, E' and A') and trans‐mitral Doppler (E, A and E/A ratio).

#### 2.3.2 Mechanics

Images were acquired and optimized for myocardial speckle tracking. This involved maintaining frame rates between 67 and 90 frames⋅s^−1^, depth to ensure adequate imaging of the chamber of interest and compression and reject to ensure endocardial delineation. The RV focused view and the apical two chamber, four‐chamber and long‐axis view were utilized for RV and LV GLS, respectively. For both the RV and LV views the myocardium was manually traced to include the septum and adjusted so that the region of interest (ROI) incorporated all of the wall thickness while avoiding the pericardium. Average RV GLS curves were generated as an average of the three segments of the RV free wall. LV GLS and time derivative strain rate was obtained by averaging the single strain measurements of the three separate apical LV views. If inappropriate tracking of segments was observed visually or detected by the system, retracing was performed until all segments were considered acceptable for analysis.

### 2.4 Cardiac autonomic modulation

The Task Force® Monitor (TFM) (CNSystems, Graz, Austria) is a validated monitoring system that provides continuous non‐invasive beat‐to‐beat monitoring and automatic online calculation of haemodynamic and cardiac autonomic (HRV) parameters (Fortin et al., 1998; Valipour et al., 2005). The TFM enables the continuous measurement of BP using the vascular unloading technique (Fortin et al., 1998; Gratze et al., 1998), which is automatically corrected to oscillometric BP values obtained at the brachial artery of the contralateral arm. A 6‐channel ECG is included for R‐R interval determination (Valipour et al., 2005) and the beat‐to‐beat values are used for the real‐time calculation of HRV by an autoregressive model (Bianchi et al., 1997) and are displayed as 3‐dimensional sliding power spectra. To obtain autonomic functional measures, participants were supine in a quiet, darkened room for 15‐min^−1^ followed by a 5‐min recording for data analysis. The 5‐min recording was repeated following the UD trial and between each exercise component in the BD trial.

### 2.5 Blood sampling

Venous cannulation was performed immediately after the TTE and autonomic assessment, immediately prior to exercise to reduce any confounding influence on autonomic function and resting BP. Three 5 mL samples of venous blood were withdrawn into heparinised vacutainers via cannulation of the cephalic vein. Samples were then centrifuged at 1500 rpm for 15‐min^−1^ to separate the blood plasma (Giavarina & Lippi, 2017). Plasma was carefully extracted via pipet into 2 mL storage vials, which were labeled with a code to protect the sample identity and placed in purpose designed plastic storage containers within a securely padlocked freezer at −80°C at Canterbury Christ Church University. The remaining cell matter was disposed of via incineration at a biomedical waste centre. Once all samples had been collected, plasma vials were transported via specialist courier to St George's University Hospitals NHS Foundation Trust, London, where they were subsequently analyzed for hs‐cTnT (Roche).

### 2.6 Statistical analysis

#### 2.6.1 Sample size calculation

Sample size determination was performed using G*Power (Version 3.1.9.7, Heinrich‐Heine‐Universität Düsseldorf, Germany). Based on previous research examining post‐exercise reductions in left ventricular longitudinal strain following endurance exercise (References), we anticipated a 20% reduction in global longitudinal strain. Using an *α* = 0.05, *β* = 0.80, and an expected effect size (*d*) of 1.2 calculated from previous studies examining cardiac strain following prolonged exercise, a minimum sample size of 8 participants was required for a paired *t*‐test analysis. To account for potential dropout and technical issues with cardiac imaging, we recruited 10 participants. This sample size aligns with previous investigations in the field examining cardiac fatigue in endurance athletes (Shave et al., 2004).

Unless otherwise stated, continuous variables are expressed as mean ± standard deviation (SD). All data were analyzed using the statistical package for social sciences (SPSS 22 release version for Windows; SPSS Inc., Chicago IL, USA). Data were assessed for conformity with parametric assumptions (Field, 2000). A repeated measures analysis of variance (ANOVA) was performed, followed by Bonferroni post hoc tests for multiple comparisons (Field, 2000). To explore the relationship between individual variations in exercise intensity, autonomic modulation and cardiac functional changes we performed linear correlations on the pre to post‐exercise changes in RMSSD with three other variables (LV GLS, hs‐cTnT release and average exercise heart rate) using the Scikit‐learn python package (Pedregosa et al., 2011). A *p* value of <0.05 was regarded as statistically significant.

## 3 RESULTS

### 3.1 Exercise results

The average time to complete the legs of each duathlon were not significantly different between BD and UD trials (Run 1: 41.9 ± 1.9 vs. 42.0 ± 2.8 min^−1^, Bike: 65.1 ± 4.2 vs. 67.8 ± 3.9 min^−1^, Run 2: 21.3 ± 1.6 vs. 21.7 ± 2.1 min^−1^, total time 128.3 ± 6.0 vs. 131.5 ± 7.0 min^−1^ for BD and UD, respectively). Additionally, there were no significant differences in mean *V* O_2_ (Run 1: 53.3 ± 4.4 vs. 54.0 ± 4.8 mL⋅kg^−1^⋅min^−1^, Bike: 44.0 ± 6.0 vs. 43.6 ± 6.6 mL⋅kg^−1^⋅min^−1^, Run 2: 52.1 ± 5.4 vs. 52.7 ± 5.4 mL⋅kg^−1^⋅min^−1^) or HR (Run 1: 171.9 ± 11.7 vs. 169.3 ± 12.7 b⋅min^−1^, Bike: 154.7 ± 11.5 vs.151.5 ± 11.9 b⋅min^−1^, Run 2: 172.9 ± 9.3 vs. 168.8 ± 11.4 b⋅min^−1^). Cycling power output was also similar between BD and UD trials (210.6 ± 38.2 vs. 200.8 ± 32.7 W). Detailed results of exercise and physiological parameters at each timepoint can be found in the Table S1.

#### 3.1.1 Cardiac function

In both trials there were immediate post‐exercise alterations in cardiac function attributed to loading conditions, including increased cardiac output (*Q*˙) (UD F (2, 18) = 9.30, *p* = 0.001, *η* ^2^ *p* = 0.51, 95% CI [4.2, 7.8 L/min]; BD F (4, 36) = 4.62, *p* = 0.004, *η* ^2^ *p* = 0.34, 95% CI [3.9, 7.2 L/min]), reduced mitral E/A ratio (UD F (2, 18) = 15.02, *p* < 0.001, *η* ^2^ *p* = 0.63, 95% CI [−0.8, −0.3]; BD F (4, 36) = 7.59, *p* < 0.001, *η* ^2^ *p* = 0.46, 95% CI [−0.7, −0.2]), reduced tricuspid E/A ratio (UD F (2, 18) = 15.29, *p* < 0.001, *η* ^2^ *p* = 0.67; BD F (4, 36) = 7.72, *p* < 0.001, *η* ^2^ *p* = 0.46) (Table 2). Elevations in HR (UD F (2, 18) = 3.71, *p* < 0.001; BD F (4, 36) = 2.89, *p* < 0.001), were correlated with changes in systolic and diastolic cardiac function. Individual repeated measures correlation analyses found moderate to strong correlations between HR and *Q*˙ (UD *r* = 0.71, 95% CI [0.40,0.87], *p* = 0.003; BD *r* = 0.53, 95% CI [0.27,0.72], *p* < 0.001), and HR with E/A ratio (UD *r* = −0.80, 95% CI [−0.91, −0.56], *p* = 0.002; BD *r* = −0.48, 95% CI [−0.69, −0.2], *p* = 0.004). In the BD trial there were also post‐exercise reductions in EDV (F (4, 36) = 3.69, *p* = 0.012, *η* ^2^ *p* = 0.29, 95% CI [−15.2, −5.8 mL]) and SV (F (4, 36) = 4.54, *p* = 0.041, *η* ^2^ *p* = 0.33).

**TABLE 2:** Left and right ventricular PW Doppler and TDI measures at each timepoint.

	Broken duathlon					Unbroken duathlon		
	Pre	Run 1	Bike	Post	24hPost	Pre	Post	24 h post
Left Ventricle								
E (cm/s)	0.70 (0.12)	0.66 (0.09)	0.64 (0.11)	0.59 (0.13)	0.7 (0.15)	0.75 (0.17)	0.70 (0.1)	0.76 (0.11)
A (cm/s)	0.40 (0.07)	0.49 (0.12)	0.47 (0.09)	0.45 (0.06)	0.39 (0.11)	0.42 (0.07)	0.55 (0.11)*	0.43 (0.07)^‡^
E/A Ratio	1.78 (0.23)	1.43 (0.39)*	1.44 (0.34)	1.34 (0.35)*	1.93 (0.59)	1.80 (0.38)	1.31 (0.23)*	1.79 (0.22)^‡^
E/E'	4.12 (1.0)	4.0 (0.72)	4.21 (0.9)	3.88 (1.03)	4.38 (0.79)	4.35 (0.97)	4.54 (1.1)	4.39 (1.18)
Right Ventricle								
TAPSE (cm)	2.8 (0.3)	2.6 (0.3)	2.7 (0.4)	2.6 (0.4)	2.8 (0.3)	2.9 (0.3)	2.8 (0.3)	2.7 (0.4)
E (cm/s)	0.68 (0.06)	0.52 (0.06)*	0.54 (0.07)	0.51 (0.03)*	0.62 (0.10)	0.72 (0.11)	0.58 (0.12)*	0.69 (0.11)
A (cm/s)	0.32 (0.01)	0.31 (0.02)	0.33 (0.03)	0.39 (0.08)*	0.32 (0.03)^‡^	0.36 (0.08)	0.44 (0.13)*	0.33 (0.03)
E/A Ratio	1.8 (0.3)	1.6 (0.3)*	1.6 (0.4)	1.3 (0.2)*	1.9 (0.4)^‡^	2.1 (0.3)	1.3 (0.2)*	2.0 (0.3)^‡^

*Note*: *n* = 10 participants. Paired‐samples *t*‐tests results.

* Significantly (*p* = 0.05) different from within trial baseline measurement.

^‡^ Significantly (*p* < 0.05) different from within trial post exercise measurement.

#### 3.1.2 Cardiac mechanics

**FIGURE 2:**
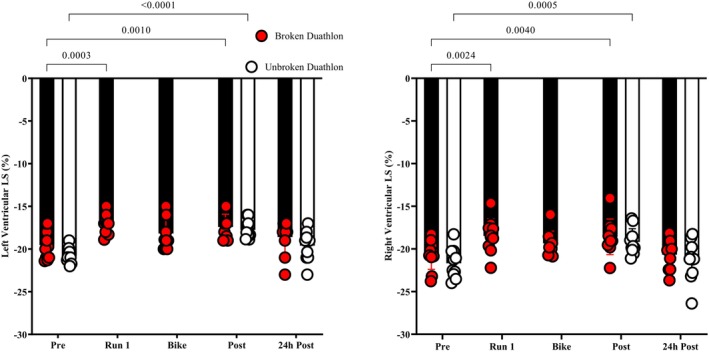
Plot of left and right ventricular LS at each time point in both trials. *n* = 10 participants. Results of the paired‐samples *t*‐tests results are presented above the figures where significant (*p* = 0.05) differences from the pre‐exercise values within each trial occurred. Individual data points are represented by red circles for the BD trials and open circles for the UD trials.

Left ventricular GLS was reduced post‐exercise in both duathlons (UD F (2, 18) = 3.92, *p* = 0.038, *η* ^2^ *p* = 0.29, 95% CI [−22.1, −18.2%]; BD F (4, 36) = 4.41, *p* = 0.005, *η* ^2^ *p* = 0.33, 95% CI [−21.8%, −17.9%]), as well as RV GLS (UD F (2, 18) = 4.23, *p* = 0.003, *η* ^2^ *p* = 0.32, 95% CI [−24.3%, −19.8%]; BD F (4, 36) = 5.91, *p* = 0.002, *η* ^2^ *p* = 0.40, 95% CI [−23.9%, −19.2%]) (Figure 2). The baseline to immediate post‐exercise changes in RV GLS in both duathlon trials were correlated with alterations in LV deformation rate (early diastolic SR: *r* = −0.44, 95% CI [−0.64, −0.19], *p* = 0.003; systolic SR: *r* = 0.41, 95% CI [0.15, 0.61], *p* = 0.004). Additionally, LV late diastolic SR was increased (UD F (2, 18) = 17.70, *p* = 0.002, *η* ^2^ *p* = 0.66; BD F (4, 36) = 4.94, *p* = 0.003, *η* ^2^ *p* = 0.35) (Table 3).

**TABLE 3:** Left and right ventricle longitudinal strain rate data from each trial.

Phase	Broken duathlon					Unbroken duathlon		
	Pre	Run1	Bike	Post	24 h post	Pre	Post	24 h post
Left Ventricle								
Systolic SR (%/s)	−1.05 (0.08)	−1.03 (0.09)	−1.05 (0.12)	−1.04 (0.09)	−1.01 (0.09)	−1.17 (0.11)	−1.23 (0.12)	−1.12 (0.1)
Early Diastolic SR (%/s)	1.79 (0.26)	1.75 (0.22)	1.79 (0.33)	1.73 (0.27)	1.69 (0.18)	1.81 (0.44)	1.87 (0.37)	1.76 (0.14)
Late Diastolic SR (%/s)	0.68 (0.16)	0.87 (0.24)*	0.95 (0.31)*	0.99 (0.34)*	0.72 (0.18)	0.78 (0.19)	1.04 (0.28)*	0.75 (0.17)
Right Ventricle								
Systolic SR (%/s)	0.88 (0.19)	1.03 (0.37)	0.89 (0.32)	1.04 (0.33)	0.80 (0.23)	0.96 (0.22)	1.01 (0.32)	0.85 (0.25)
Early Diastolic SR (%/s)	1.37 (0.38)	1.25 (0.25)	1.34 (0.43)	1.22 (0.23)	1.27 (0.18)	1.41 (0.30)	1.38 (0.26)	1.25 (0.47)
Late Diastolic SR (%/s)	−1.07 (0.18)	−1.1 (0.22)	−1.09 (0.13)	−1.05 (0.16)	−1.08 (0.14)	−1.13 (0.13)	−1.05 (0.07)	−1.33 (0.22)

*Note*: *n* = 10 participants. Global LV LS parameters (LVLS), peak longitudinal systolic ε (LVLS S), longitudinal SR for peak late diastole (LVSR A), early diastole (LVSR E), and systole (LVSR S). Paired sample *t*‐test results.

* Significantly different from baseline levels within each trial (*p* < 0.05).

Significantly (*p* < 0.05) different from post exercise measurement within each trial.

### 3.2 Haemodynamics and heart rate variability

There were post‐exercise reductions in the LF/HF ratio (F (2, 18) = 15.31, *p* < 0.001, *η* ^2^ *p* = 0.66, 95% CI [−4.2, −1.8]), RMSSD (F (2, 18) = 39.30, *p* < 0.001, *η* ^2^ *p* = 0.83, 95% CI [−42.3, −28.7 ms]), total PSD (UD F (2, 18) = 5.62, *p* = 0.014, *η* ^2^ *p* = 0.41, 95% CI [−8234, −3567 ms^2^]; BD F (4, 36) = 5.76, *p* = 0.001, *η* ^2^ *p* = 0.39, 95% CI [−7856, −3124 ms^2^]), and RMSSD (F (4, 36) = 5.92, *p* = 0.001, *η* ^2^ *p* = 0.40, 95% CI [−38.5, −25.2 ms]). In addition, mean arterial pressure (F (2, 18) = 4.61, *p* = 0.026, *η* ^2^ *p* = 0.37, 95% CI [−8.2, −2.4 mmHg]) and diastolic BP (F (2, 18) = 5.85, *p* = 0.012, *η* ^2^ *p* = 0.42, 95% CI [−7.8, −2.1 mmHg]) were lower than pre‐exercise measurements after 24 h^−1^ of recovery. LF/HF ratio was also lower than baseline in the UD trial, but this was reversed following 24 h^−1^ of rest. In the BD trial, RMSSD, and PSD were reduced following Run 1 and remained significantly lower than baseline following each leg of the duathlon until the 24 h^−1^ recovery point (Table 4). The results of the linear regression demonstrated significant relationship between changes in RMSSD (ΔRMSSD) and LV GLS, average exercise HR, and troponin release (Table 5).

**TABLE 4:** Haemodynamic and log‐transformed HRV variables for each duathlon trial and timepoint.

	Broken duathlon					Unbroken duathlon		
	Pre	Run 1	Bike	Post	24 h post	Pre	Post	24 h post
Haemodynamics								
sBP (mmHg)	115.5 (11.4)	103.7 (9.6)	107.0 (9.4)	121.2 (9.3)	117.0 (6.5)	115.7 (15.4)	122.2 (9.6)	111.6 (16.7)
mBP (mmHg)	86.5 (10.0)	77.8 (29.6)	83.2 (8.0)	91.9 (6.3)^×^	86.2 (4.6)	86.3 (12.6)	91.1 (7.4)	79.7 (11.0)^‡^
dBP (mmHg)	68.2 (8.0)	60.3 (12.9)	67.6 (7.7)	73.0 (5.1)	67.6 (5.0)	68.0 (10.9)	72.1 (6.6)	61.8 (8.2)^‡^
Heart Rate Variability								
RRI (ms)	7.04 (0.14)	6.49 (0.09)	6.45 (0.69)	6.55 (0.1)	7.03 (0.13)	7.11 (0.18)	6.58 (0.17)*	6.97 (0.16)*, ^‡^
RMSSD (ms)	60.31 (16.39)	28.71 (23.88)*	32.86 (18.04)*	32.96 (29.69)*	55.58 (22.06)^‡^	82.35 (22.36)	17.41 (9.71)*	49.67 (19.26)*, ^‡^
PSD (ms)	8.18 (0.73)	5.25 (1.67)*	7.03 (1.04)*	5.96 (1.77)*	7.93 (0.89)^‡^	8.72 (0.81)	5.33 (1.97)*	7.68 (0.91)^‡^
Hfnu (ms)	56.58 (11.53)	64.41 (18.36)	66.74 (20.98)	63.97 (24.52)	57.6 (13.33)	51.58 (10.37)	80.85 (10.12)	54.15 (11.79)
Lfnu (ms)	43.46 (11.5)	29.48 (18.5)	31.65 (21.16)	27.01 (12.01)	42.4 (13.33)	48.42 (10.37)	18.7 (10.24)	45.72 (11.9)
LF/HF	7.2 (0.7)	4.47 (1.5)	6.3 (0.98)	5.18 (1.67)	6.96 (0.85)	7.69 (0.78)	4.86 (2.05)*	6.68 (0.85)^‡^

*Note*: *n* = 10 participants. Paired samples *t*‐test.

Abbreviations: dBP, diastolic blood pressure; mBP, mean arterial blood pressure; sBP, systolic blood pressure.

* Significantly (*p* < 0.05) different from baseline measurement within each trial.

^‡^ Significantly different from post exercise measurement within each trial.

**TABLE 5:** Univariate linear correlation results for ΔRMSSD vs. mean exercise HR, LV longitudinal strain, and hs‐cTnT release from both duathlon trials.

Variable	*β*	CI [2.5%]	CI [97.5%]	SE	T	*p*	*R* ^2^
LV LS	−0.63	−0.54	−0.53	0.05	−12.15	0.001	0.39
hs‐cTnT (ug∙L^−1^)	−0.40	−0.52	−0.28	−0.06	−6.42	0.001	0.45
HR_AVG_	−3.55	−5.14	−1.96	0.76	−4.70	0.00	0.55

*Note*: *n* = 20 observations, 10 participants. Linear correlation results.

Abbreviations: *β*, beta coefficient; CI, confidence interval; HR_AVG_, average HRs during both duathlons; HR_PEAK_, peak HR during the duathlon; SE, standard error.

### 3.3 Cardiac troponin

**FIGURE 3:**
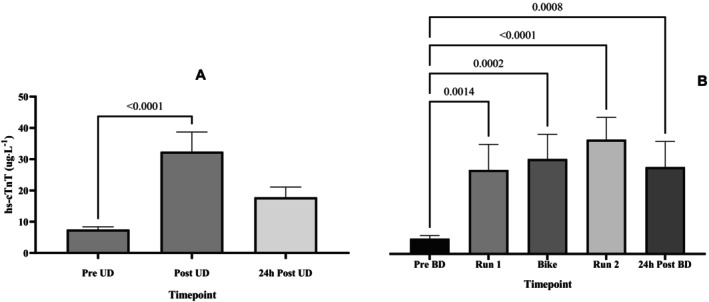
Bar plot of mean hs‐cTnT concentrations after each leg of the UD trial (a) and the UD trial (b), *p* values are displayed compared to pre‐duathlon levels.

The hs‐cTnT was elevated at the end of exercise in both trials (Figure 3). In the BD trial, mean hs‐cTnT rose above baseline after Run 1 and the concentration increased over the subsequent 2 legs (*p* < 0.03). At the 24 h^−1^ recovery TP there were still elevations above baseline in hs‐cTnT in both trials (Figure 3).

## 4 DISCUSSION

### 4.1 Key findings

This study was the first to examine cardiac function and regulation following OD duathlon exercise. In line with the aims, the key findings were that the cardiac systems of highly trained triathletes recover from ~2 h of strenuous duathlon exercise within 24 h, and that subsequent race performance within 7 days was not affected by any lingering cardiac fatigue. We also demonstrated that experienced, fit triathletes are capable of reproducing self‐paced OD duathlon performances at target race‐pace within the laboratory environment. In terms of cardiac functional changes, we observed that transient reductions in longitudinal strain and autonomic balance began during or immediately after the initial 10 km run, and some recovery was seen in longitudinal function following the bike leg, which returned to post 10 km run levels following the 5 k run. This accompanied significant release of hs‐cTnT, which continued to increase throughout the legs of the duathlon. Like the majority of exercise induced cardiac functional change research, we observed almost total reversal or amelioration of all changes within 24 h^−1^ of passive recovery (Stewart et al., 2014, 2016).

### 4.2 Exercise results

The exercise performances achieved by the participants were like those achieved in previous works investigating the physiological and metabolic effects of duathlon performances (Berry et al., 2016; Sparks et al., 2005). In each leg of both duathlon trials, participants were able to sustain a high percentage of their *V* ^⋅^O_2max_ and max HRs in both sports, as they would under typical race conditions (Millet et al., 2011). In both run legs, participants demonstrated higher *V* ^⋅^O_2_, mean HRs, and peak HRs than during the bike legs. This finding corroborates the work of Kohrt and colleagues, who demonstrated significantly higher *V* ^⋅^O_2max_ in treadmill vs. cycling CPET (Kohrt et al., 1987). However, in amateur level triathletes, *V* ^⋅^O_2max_ tends to be similar between the two exercise modes (Miura & Ishiko, 1994), which suggest that the cycling leg in the current study were completed at lower relative intensities. In contrast to this, Schabort and colleagues found that running *V* ^⋅^O_2max_ was significantly higher than cycling in national‐level triathletes (68.9 vs. 65.6 mL∙kg∙min^−1^) (Schabort et al., 2000). Based on *V* ^⋅^O_2max_ data the participants in the current study were more typical of national‐level triathletes and this may explain the disparity in the strenuousness of the running versus cycling legs (Millet et al., 2011).

The overall lower intensity during the bike than the run legs may have been a result of the influence of initial reductions in cardiac function reported after the first run. In elite triathlon (and duathlon) competition, the bike leg requires a more varied, stochastic power output, with periods of high‐intensity anaerobic effort. Therefore, this limits the inference of the results of this study to draft‐legal triathlon or duathlon performance.

In terms of recovery between the two duathlon performances, we found that there were no significant differences within participants between duathlon trials for the times to complete each leg, average HRs, gas exchange data, cycling power outputs and run speed during each leg. There has been substantial debate as to whether EICF effects progressively build over time to cause impact on cardiovascular function, and whether athletes and coaches need to consider the ‘strain’ of EICF as part of their recovery. These data demonstrate there were no effects of the prior duathlon and subsequent EICF on subsequent performance or cardiac function. We chose to use a rest period of 7 days as this represents the typical seasonal race frequency of competitive triathletes. Whether more‐frequent racing elicits a progressive decline in cardiac function and exercise performance is unknown.

### 4.3 Echocardiographic results

#### 4.3.1 Ventricular systolic and diastolic function

The immediate effects of PSE during the duathlons were an increased HR, reduced EDV, increased *Q*˙ and reduced mitral and tricuspid E/A ratio due to reductions in early and increases in late trans‐mitral filling velocities. These findings align with existing literature on post‐exercise cardiac function (Donaldson et al., 2019; Lord et al., 2018; Middleton et al., 2006). While some studies have reported acute alterations in systolic function following prolonged exercise (Alshaher et al., 2007; Dawson et al., 2005; George et al., 2004; Hart et al., 2007; Lucia et al., 1999; Neilan et al., 2006), others have found systolic function to be preserved (Middleton et al., 2007; Shave et al., 2004), suggesting the response may be exercise‐mode dependent.

The post‐exercise reductions in mitral E/A ratio were strongly associated with elevations in HR (Middleton et al., 2007). Elevated HR is thought to play a potentially confounding role in diagnosing changes in LV diastolic function due to reductions in ventricular relaxation time (Oniki et al., 1992). This was supported by the findings of reduced E velocity and significant correlations between HR and mitral valve PW variables. However, there is evidence to suggest that reductions in diastolic function occur in the absence of positive correlations to HR and preload (Middleton et al., 2006). In the BD trial, the magnitude of the change in E/A ratio increased with each leg of the duathlon due to progressively lower E velocities. A possible explanation for this may be that the total cumulative duration of the exercise was the cause of altered diastolic function. This would support the findings of previous meta‐analyses that determined impairments in diastolic function become more prolific following longer‐duration exercise (Middleton et al., 2006; Oxborough et al., 2010; Shave & Oxborough, 2012).

In terms of systolic function, *Q*˙ increased alongside HR while SV and EF reduced following each leg of the duathlon in the BD trial. Despite being able to increase overall cardiac output, both run legs yielded small reductions in EDV, which contributed to the loss of SV. The transient nature of these alterations, with values returning toward baseline during the bike leg, suggests that any systolic dysfunction was temporary rather than indicative of persistent cardiac fatigue. This pattern of recovery during cycling was also reflected in the GLS data.

#### 4.3.2 Ventricular global longitudinal strain

The finding of decreased left and right ventricular GLS following each duathlon trial is in line with previous research (Lord et al., 2018). During the BD trial, LV GLS was significantly lower than baseline following the first run then recovered after the bike to near pre‐exercise values. During the bike leg, participants averaged lower exercise HRs and *V* ^⋅^O_2_, therefore despite the pre‐completed run exercise, participants in this study were able to recover their ventricular longitudinal function during the bike trial. The steady state nature of cycling exercise may have facilitated this recovery. Previous research by Stewart et al. (2015) demonstrated transient reductions in LV GLS to a similar level (~1%) following a criterium style race of equal time to the bike leg in this study (~60 min^−1^), which required a stochastic power output demand from the participants (Stewart et al., 2015). The bike leg in the present study was steady state in nature to replicate the non‐drafting demands of duathlon racing, which may have required less cardiac work; however, the cardiac fatigue differences between running and cycling are currently unclear. The finding of a return to below BL levels in LV GLS following the second run adds further support to the emerging theory that running exercise places a greater level of cardiac demand than does cycling exercise. Unfortunately, measurements of atrial strain were not possible in this study; however, the effect of a disproportionate level of atrial strain over LV GLS cannot be ruled out (Saraiva et al., 2010; Sareban et al., 2018).

Our findings of concomitantly reduced RV longitudinal strain support the body of evidence that suggests RV mechanics are attenuated to an equal or greater extent following exercise than the LV (Oxborough et al., 2012). This mechanism is potentially mediated by several factors including increased afterload during exercise on the RV, resulting in disproportionate haemodynamic loading of the RV (Lewicka‐Potocka et al., 2020; Lord et al., 2015). Additionally, LV deformation has been shown to impact RV strain (Oxborough et al., 2011), and previous research has demonstrated an increased incidence of septal bounce secondary to RV dilatation following PSE (Dawson et al., 2005; La Gerche et al., 2012; Oxborough et al., 2011; Oxborough et al., 2012). The data from this study confirms these previous findings as there was significant, moderate correlations between RV strain and LV diastolic and systolic strain rates.

### 4.4 Heart rate variability

Similar to the findings of Stewart et al. (2014) and Seiler et al. (2007) there was evidence of sustained sympathetic nervous system outflow and parasympathetic tone suppression during the acute recovery phase from both duathlons, measured by perturbations in LF/HF balance (Seiler et al., 2007). At rest, the LF/HF ratio, PSD and RMSSD were similar in the participant cohort to previously reported data in similar demographics (Stewart et al., 2014). In the present study, the lower RMSSD and total PSD of RR intervals during immediate recovery reflects reduced parasympathetic cardiac modulation, while the increased LF:HF ratio is reflective of sustained sympathetic outflow during the acute recovery phase from the duathlon exercise. In the BD trial, parasympathetic modulation was significantly reduced from BL following the first run, and remained suppressed until the 24 h^−1^ recovery point.

The overall findings from this study of significant reductions in post‐exercise parasympathetic modulation, myocardial longitudinal function, alongside post‐exercise troponin release raises further questions about the relationship between these aspects of cardiac function (Stewart et al., 2016). The mechanism behind this response is still unclear; however, Stewart and colleagues have proposed that the upper limit of the moderate‐intensity exercise domain (given as the gas exchange threshold (GET) in their study) demarcates a threshold for EICF and represents an intensity boundary that influences both cardiac health and adaptation (Pringle et al., 2003). In their study, the participants exercised at two intensities, above or below the GET for 90 and 120 min^−1^, respectively (Stewart et al., 2016). The majority of cardiac autonomic and functional perturbations occurred following the high‐intensity 90‐min bout of exercise, above the proposed intensity boundary. In the present study, average relative HR and work rates were similar to the ‘high‐intensity’ bout in the study of Stewart and colleagues (Stewart et al., 2016), despite participants self‐selecting their effort levels.

The finding of moderate associations between exercise heart rates, LVGLS alterations, and hs‐cTnT release with RMSSD supports the potential existence of an individual threshold of post‐exercise transiently altered haemodynamic and autonomic function which demarcates the onset of EICF, which has been evidenced recently (Coates et al., 2023). This finding demonstrates the influence of post‐exercise haemodynamic and vagal alterations on time‐domain analysis of HRV as a non‐invasive marker for exercise recovery.

### 4.5 Cardiac biomarkers

Similar to the majority of research in this field (Donaldson et al., 2019; Lord et al., 2018; Shave & Oxborough, 2012), it was shown that duathlon performance elicits significant elevations in cardiac troponin biomarkers, which were reversed within 24 h^−1^ of recovery in the UD trial but sustained above pre‐exercise levels in the BD trial. It is important to note that the mean concentrations of troponin release did not exceed clinical thresholds for diagnosis of myocardial infarction. Further, in the event of myocardial ischaemia it has been demonstrated that hs‐cTnT is detectable in a bi‐phasic pattern over a 48 h period with an initial peak thought to represent the release of free troponin from the cytosolic pool and a subsequent peak and sustained elevation caused by necrosis of the myocardium and leakage of the contractile proteins into the blood stream (Shave, Baggish, et al., 2010).

### 4.6 Limitations

The confounding influence of increased post‐exercise HRs and loading condition alterations that would have undoubtedly affected the reported conventional LV systolic should not be overlooked. By performing a stress‐echo to achieve a target HR (i.e., 100 b·min^−1^) during the post‐exercise measures this could have been accounted for and has now been implemented in several studies. Owing to methodological and equipment limitations we were unable to utilize stress echo in the present study (Coates et al., 2023; Stewart et al., 2015; Stewart et al., 2016). In addition, our study only included male athletes, which limits the generalizability of our findings to female athletes and other populations.

### 4.7 Conclusion

At the time of writing, this was the first study conducted that examined EICF following Olympic‐distance duathlon performance. The results have demonstrated that reproducible duathlon performances in the laboratory environment are possible by highly trained, experienced triathletes. While laboratory conditions offer high internal validity for measuring physiological responses, we acknowledge that environmental factors such as terrain, temperature, and wind resistance in outdoor competitions may influence the physiological demands. Previous field studies have shown similar cardiovascular responses between laboratory and outdoor multisport events (Berry, 2012; Nikolaidis et al., 2019, 2021; Rust et al., 2013; Sparks et al., 2005), though direct comparisons warrant further investigation. The absence of any significant differences in exercise VO₂ and HR between trials, despite the additional 30‐min measurement period between legs in the broken duathlon trial, has demonstrated the feasibility of future broken multisport trials to investigate the development of EICF.

Our results demonstrate that Olympic‐distance duathlon performance produces similar elevations in cardiac biomarkers, autonomic regulatory changes, and transient reductions in cardiac systolic and diastolic functional measures as previously evidenced in triathlon, and standalone running or cycling performance. Importantly, there were no significant differences in exercise performance or the acute magnitude of transient cardiac functional, biomarker and regulatory perturbations between continuous and discontinuous exercise trials, and no lingering effects of the previous trial during the second duathlon performance.

These findings have several practical implications for athletes and coaches:

1. The similar cardiac responses between continuous and broken protocols suggest that either format could be used effectively in training.
2. The transient nature of the cardiac alterations indicates that appropriate recovery between such events is achievable.
3. The cardiac strain appears manageable compared to ultra‐endurance events, informing recovery strategies.

Future research directions should:

1. Conduct field‐based studies to validate these laboratory findings in various environmental conditions.
2. Investigate the minimum recovery time needed between high‐intensity multisport events.
3. Examine individual variability in cardiac responses to inform personalized training approaches.

## FUNDING INFORMATION

No funding information provided.

## Supporting information


Data S1.
