# Supplementary material for: Olympic distance duathlon and cardiac performance in highly‐trained triathletes
Source: Physiol Rep. 2024 Dec 26;12(24):e70154. doi: 10.14814/phy2.70154 (PMC11671242; doi:10.14814/phy2.70154)
Supplement: Supplementary file 1 — Data S1. [file PHY2-12-e70154-s001.docx]

Supplemental Table 1. Exercise data at each intra-leg timepoint (TP). Note: There were no significant between-trial differences between exercise performance variables at each TP.

| Trial | | | | | | | Broken Duathlon | | | | | | | |
| --- | --- | --- | --- | --- | --- | --- | --- | --- | --- | --- | --- | --- | --- | --- |
| Run1 (10k) | | | | | | | Bike (40k) | | | | Run2 (5k) | | | |
| Timepoint | Run 1 - 1 | Run 1 - 2 | Run 1 – 3 | Run 1 – 4 | Bike – 1 | Bike – 2 | | Bike – 3 | Bike – 4 | Bike – 5 | | Bike – 6 | Run 2 – 1 | Run 2 - 2 |
| Time (minutes) | 10 | 20 | 30 | 35 | 10 | 20 | | 30 | 40 | 50 | | 55 | 10 | 15 |
| HR beats∙min^-1^ | 165.4 (13.1) | 171.2 (13) | 174.2 (9.4) | 176.9 (9) | 150.4 (10.2) | 153.3 (12.3) | | 154.5 (12.6) | 156.5 (12.3) | 156.4 (12.3) | | 157.2 (10.6) | 170.1 (9.8) | 175.8 (8.4) |
| Run Speed (km∙hour^-1^) | 14.3 (0.7) | 14.3 (0.7) | 14.4 (0.8) | 14.5 (0.9) | - | - | | - | - | - | | - | 14.1 (1) | 14.4 (1.5) |
| *V*⋅O_2_ (L∙min^-1^) | 3.9 (0.3) | 3.9 (0.3) | 4 (0.3) | 4 (0.4) | 3.2 (0.4) | 3.3 (0.5) | | 3.2 (0.5) | 3.3 (0.4) | 3.3 (0.5) | | 3.3 (0.3) | 3.8 (0.3) | 3.9 (0.4) |
| *V*⋅O_2_ (mL∙kg∙min^-1^) | 52.6 (4.5) | 53.2 (4.7) | 53.5 (4.3) | 53.8 (4.7) | 42.8 (6.1) | 44.5 (5.8) | | 43.6 (6.9) | 44.3 (6.2) | 44.5 (6.9) | | 44.6 (5.6) | 51.4 (4.9) | 52.8 (6) |
| Watts | **-** | - | - | - | 205.1 (41.3) | 216 (39.4) | | 208.7 (42) | 213.3 (39.2) | 208.6 (38.1) | | 211.9 (38.5) | - | - |

| Trial | | | | | | | Unbroken Duathlon | | | | | | | | |
| --- | --- | --- | --- | --- | --- | --- | --- | --- | --- | --- | --- | --- | --- | --- | --- |
| Run1 (10k) | | | | | | | Bike (40k) | | | | Run2 (5k) | | | | |
| Timepoint | Run 1 - 1 | Run 1 - 2 | Run 1 – 3 | Run 1 – 4 | Bike – 1 | Bike – 2 | | Bike – 3 | Bike – 4 | Bike – 5 | | Bike – 6 | Run 2 – 1 | Run 2 - 2 |  |
| Time (minutes) | 10 | 20 | 30 | 35 | 10 | 20 | | 30 | 40 | 50 | | 55 | 10 | 15 |  |
| HR beats∙min^-1^ | 159.5 (9.8) | 169.3 (10.6) | 173.9 (13) | 174.6 (12.6) | 153.5 (12.7) | 152.2 (13.5) | | 150.2 (14) | 149 (12.1) | 151.1 (11.4) | | 152.9 (9.7) | 166.8 (13) | 170.9 (9.7) |  |
| Run Speed (km∙hour^-1^) | 14.3 (0.9) | 14.5 (0.9) | 14.4 (1.1) | 14.3 (1.1) | - | - | | - | - | - | | - | 14.1 (1.1) | 11.1 (6) |  |
| *V*⋅O_2_ (L∙min^-1^) | 3.9 (0.3) | 4 (0.3) | 4 (0.4) | 3.9 (0.4) | 3.1 (0.5) | 3.1 (0.5) | | 3.1 (0.5) | 3.2 (0.5) | 3.3 (0.4) | | 3.3 (0.5) | 3.8 (0.4) | 3.9 (0.3) |  |
| *V*⋅O_2_ (mL∙kg∙min^-1^) | 53.8 (4.5) | 54.2 (5) | 54.7 (5.4) | 53.4 (5.1) | 42.4 (7.2) | 42.5 (6.9) | | 43.1 (6.7) | 43.8 (7.2) | 44.8 (6.4) | | 45.4 (6.4) | 52.3 (5.4) | 53 (5.6) |  |
| Watts | - | - | - | - | 194.1 (35.5) | 195.7 (29) | | 196.9 (33.9) | 201 (33.4) | 205.2 (33.2) | | 211.6 (35.7) | - | - |  |
